# Biosynthesized silver nanoparticles mediated by *Ammi visnaga* extract enhanced systemic resistance and triggered multiple defense-related genes, including SbWRKY transcription factors, against tobacco mosaic virus infection

**DOI:** 10.1186/s12870-024-05449-y

**Published:** 2024-08-07

**Authors:** Dalia G. Aseel, Omar M. Ibrahim, Ahmed Abdelkhalek

**Affiliations:** 1 Plant Protection and Biomolecular Diagnosis Department, Arid Lands Cultivation Research Institute (ALCRI), City of Scientific Research and Technological Applications (SRTA-City), New Borg El-Arab City, 21934 Egypt; 2 Plant Production Department, Arid Lands Cultivation Research Institute (ALCRI), City of Scientific Research and Technological Applications (SRTA-City), New Borg El-Arab City, 21934 Egypt

**Keywords:** *Ammi visnaga*, Ag-NPs, Antiviral activity, TEM, FTIR, Defense-related gens expression

## Abstract

**Background:**

Tobacco mosaic virus (TMV) is a highly infectious plant virus that affects a wide variety of plants and reduces crop yields around the world. Here, we assessed the effectiveness of using *Ammi visnaga* aqueous seed extract to synthesize silver nanoparticles (Ag-NPs) and their potential to combat TMV. Different techniques were used to characterize Ag-NPs, such as scanning and transmission electron microscopy (SEM, TEM), energy-dispersive X-ray spectroscopy (EDS), fourier transform infrared spectroscopy (FTIR), and dynamic light scattering (DLS).

**Results:**

TEM demonstrated that the synthesized Ag-NPs had a spherical form with an average size of 23–30 nm and a zeta potential value of -15.9 mV, while FTIR revealed various functional groups involved in Ag-NP stability and capping. Interestingly, the Pre-treatment of tobacco plants (protective treatment) with Ag-NPs at 100–500 µg/mL significantly suppressed viral symptoms, while the Post-treatment (curative treatment) delayed their appearance. Furthermore, protective and curative treatments significantly increased chlorophyll a and b, total flavonoids, total soluble carbohydrates, and antioxidant enzymes activity (PPO, POX and CAT). Simultaneously, the application of Ag-NPs resulted in a decrease in levels of oxidative stress markers (H_2_O_2_ and MDA). The RT-qPCR results and volcano plot analysis showed that the Ag-NPs treatments trigger and regulate the transcription of ten defense-related genes (*SbWRKY-1*, *SbWRKY-2*, *JERF-3*, *GST-1*, *POD*, *PR-1*, *PR-2*, *PR-12*, *PAL-1*, and *HQT-1*). The heatmap revealed that *GST-1*, the primary gene involved in anthocyanidin production, was consistently the most expressed gene across all treatments throughout the study. Analysis of the gene co-expression network revealed that *SbWRKY-1*9 was the most central gene among the studied genes, followed by *PR-12* and *PR-2*.

**Conclusions:**

Overall, the reported antiviral properties (protective and/or curative) of biosynthesized Ag-NPs against TMV lead us to recommend using Ag-NPs as a simple, stable, and eco-friendly agent in developing pest management programs against plant viral infections.

## Background

Plant diseases cause significant crop yield reductions and hinder crop management advancements. Viral infections are considered detrimental to plant biosecurity due to the significant crop losses they generate worldwide [1, 2]. Plant viruses adversely impact agriculture, necessitating significant efforts to manage viral infections. Approximately 50% of newly emerging plant diseases are caused by viruses, while many agricultural practices play a role in their transmission [3, 4]. More than 700 crop species have been documented to be infected by almost 900 kinds of plant viruses [5]. Viruses affect plants differently depending on parameters such as infection duration, environmental conditions, viral strain, host species, replication rate, and virus concentration [6]. Viral infections can lead to symptoms such as mottling, leaf drying, fruit deformation, necrosis, plant stunting, and mortality under favorable conditions for the pathogen [7]. The extensive host range of the tobacco mosaic virus (TMV), with roughly 66 families, and more than 900 plant species, makes it one of the most contagious plant diseases. Infected seeds, infected farming equipment, or sick plants might physically touch one another, leading to the mechanical transmission of TMV [8, 9]. Managing TMV infection mostly involves utilizing resistant cultivars and controlling vector transmission through the widespread application of pesticides, which could potentially harm the environment and human health [10]. There is an increasing interest in utilizing biocontrol agents as eco-friendly substitutes for harmful pesticides in plant pest control to enhance long-term sustainability in agriculture and the environment [11, 12]. Diseases, such as viruses, present a challenge in treatment, making prevention the most effective approach [13]. Currently, nanoscale materials have been produced to act as antiviral, antifungal, and antibacterial agents for animals and, to a lesser degree, for plants [14, 15].

Nanotechnology has provided new opportunities to various sectors, such as the food and beverage industry, electronics, medicine, agriculture, and health care [16]. Nanoparticles are now being utilized in plant protection and disease control as an alternative to chemical pesticides, operating within a nanoscale range of 1 nm to 100 nm. Various techniques have been introduced and achieved for producing nanoparticles. The green synthesis method is gaining popularity as environmental awareness rises, providing a more eco-friendly approach to reducing the environmental impact of NPs created using traditional physical and chemical procedures [17]. Nanoparticles, when used in appropriate doses, can enhance plant growth and prevent pathogen infection [18, 19]. Furthermore, the use of Ag-NPs enhanced plant tolerance to several biotic stressors such as worms and phytopathogenic fungi [20–22]. Although nanoparticles exhibit antiviral effects, their development is still in the initial phases, and the underlying mechanisms are mostly unknown [23, 24]. The researchers proposed that silver nanoparticles (Ag-NPs) might attach to virus particles and prevent the reproduction of viral nucleic acid in plant cells. Additionally, it aids in stimulating systemic acquired resistance (SAR) and enhancing the release of reactive oxygen species (ROS), which is linked to an increase in antioxidant activity [10, 25].

It was reported that *WRKY* transcription factors significantly impact several processes, such as pollen production, hormone control, secondary metabolite biosynthesis, seed germination, and plant stress resistance [26, 27]. The *WRKY* family is an exceptional superfamily of transcription factors found exclusively in higher plants and algae. These factors are crucial for numerous life processes, especially in the face of biotic and abiotic stress [28]. Sequential cloning of the *WRKY-1*, *WRKY-2*, and *WRKY-3* genes from *Petroselinum crispum* resulted in the production of the *WRKY* transcription factors [27]. The study discovered that the WRKY protein has the ability to control a plant’s response to a disease. In this context, the current study aimed to investigate the antiviral activity and induction of systemic resistance of biosynthesized Ag-NPs using a seed extract from the *Ammi visnaga*. The produced Ag-NPs were studied using a variety of techniques, including scanning electron microscopy (SEM), transmission electron microscopy (TEM), energy-dispersive X-ray spectroscopy (EDS), zeta potential, dynamic light scattering (DLS), and fourier transform infrared spectroscopy (FTIR). The study evaluated the antiviral properties and effectiveness of Ag-NPs in inducing SAR against TMV. It also examined their impact on photosynthetic pigments, antioxidant enzymes, oxidative stress markers. Transcription levels of two *SbWRKY* transcription factors, jasmonate and ethylene-response factor-3, together with five defense-related pathway genes and TMV accumulation levels, were analyzed to identify potential defense mechanisms.

## Materials and methods

### Source of plant materials and TMV isolate

The seeds of the *Ammi visnaga* L. plant was collected from a neighboring farm in Alexandria Governorate, Egypt. *A. visnaga* was identified by Prof. Dr. El-Sayed F. El-Halwany, Professor of Plant Ecology, Botany Department, Faculty of Science, Mansoura University, Egypt. The Egyptian agriculture research center generously contributed the virus-free tobacco seedlings (*Nicotiana tabacum* L.). The TMV strain KH1 (Acc# MG264131), which was previously obtained from infected tomato plants, is used as a viral source [29].

### Green synthesis of silver nanoparticles

Ag-NPs were synthesized utilizing a sustainable and environmentally friendly method using the reduction of silver ions with an extract from the *Ammi visnaga* seeds [10]. Before air-drying at room temperature, *A. visnaga* seeds were thoroughly rinsed three times with ultrapure Milli Q water to remove any debris or pollutants. Using a sterile electric blender, the dried seeds were processed into powder. To make the water-based extract, combine 10 g of powdered seeds with 100 mL of sterile distilled water and stir with a magnetic stirrer for two h at 60 °C. The extract mixture was filtered through sterile Whatman No. 1 filter paper and allowed to cool to room temperature. The second stage involved mixing 10 mL of seed extract with 90 mL of 1 mM AgNO_3_ solution in the dark. When the solution turns reddish-brown, it means that AgNO_3_ has been reduced and Ag-NPs are formed. The reaction liquid was centrifuged at 6000 rpm for 10 min before being washed several times with distilled water and once with 100% ethanol. After 24 h of drying at 50 °C, the precipitate was used as a source for Ag-NPs in the next assays.

### Characterization of synthesized Ag-NPs

The Ag-NPs were characterized using a range of analytical techniques. A SEM (JSM-6360 LA, JEOL, Tokyo, Japan) was used to investigate the particles’ structure and morphology at 15 KV and 5000X magnification. Furthermore, particle morphology and size were assessed using TEM on the JSM-6360 microscope (JEOL, Tokyo, Japan), and elemental analysis of Ag-NPs was conducted through the EDX unit combined with TEM [30]. The particle size analyzer (Zetasizer ver. 6.2, Malvern, UK) was used to analyze the particle size distribution and surface charge. In addition, FTIR spectroscopy (Agilent Technologies, CA, USA) was employed to assess the functional groups on the produced Ag-NPs utilizing the KBr-disc method at a wavelength range of 400–4000 cm^− 1^.

### Application of Ag-NPs under greenhouse conditions

Under greenhouse conditions, the effectiveness of foliar spraying tobacco plants with Ag-NPs to control TMV infection was assessed. A handheld pressure sprayer was used to cover every leaf on the plants. The experiment included eight treatments. The first treatment included control plants that were mechanically inoculated with viral inoculation buffer (Mock treatment). The second treatment involved plants inoculated with TMV only (TMV treatment). The third treatment contained plants treated with 100 µg/mL Ag-NPs and mechanically inoculated with viral inoculation buffer (Ag-NPs1 treatment). The fourth treatment included plants treated with 100 µg/mL Ag-NPs 48 h before TMV inoculation (Pre-1 treatment). The fifth treatment involved plants treated with 100 µg/mL Ag-NPs 48 h after TMV inoculation (Pos-1 treatment). The sixth treatment contained plants foliar treated with 500 µg/ml Ag-NPs and mechanically inoculated with viral inoculation buffer (Ag-NPs2 treatment). The seventh treatment included plants treated with 500 µg/mL Ag-NPs 48 h before TMV inoculation (Pre-2 treatment). The eighth treatment involved plants treated with 500 µg/mL Ag-NPs 48 h after TMV inoculation (Post-2 treatment). Five pots were used for each treatment, and each pot had five plants. The plants were grown for three weeks in a greenhouse that was impenetrable to insects, and we checked on them every day to see whether any symptoms had developed. The three top leaves from each plant were gathered at 18 dpi for additional analysis. The mechanical inoculation of TMV was performed as previously described [31]. The experiment was conducted in 20-cm pots containing sterilized peat moss for plant cultivation.

### Impact of Ag-NPs on physio-biochemical parameters of tobacco plants

A 2 g of harvested leaves were crushed in 5 mL of 100 mM phosphate buffer (pH 7), then centrifuged at 15,000 rpm for 20 min. The resulting supernatant was collected and utilized as a crude extract for the following physiological activity assays. The chlorophyll photosynthetic pigment was measured following the method outlined by Harborne [32]. The extraction and evaluation of the flavonoid content were conducted using the methods outlined by Jia et al. [33]. The Total soluble carbohydrate (TSC) determination was calculated following the method outlined by Islam et al. [34]. Oxidative stress indicators, such as hydrogen peroxide (H_2_O_2_) and malondialdehyde (MDA), were quantified following the methods of Junglee et al. [35] and Heath [36], respectively. Antioxidant enzyme activities were assessed by measuring polyphenol oxidase (PPO) following the method described by Duan et al. [37]. Catalase activity was determined following the method described by Aebi [38]. Peroxidase (POX) activity was assessed as described by Angelini et al. [39].

### Impact of Ag-NPs on TMV accumulation level and transcriptional levels of defense-related genes

#### Total RNA extraction and cDNA synthesis

The total RNA was extracted from 100 mg of fresh tobacco leaves using the guanidium isothiocyanate method, with minor modifications [40]. A one µg of RNA treated with DNase was used to synthesize cDNA through using M-MuLV Reverse Transcriptase [41]. A thermal cycler was utilized to conduct the reverse transcriptase reaction at 40 °C for 90 min and then deactivate it at 90 °C for 5 min.

#### Real-time quantitative PCR (RT-qPCR) assay and data analysis

The RT-qPCR method was utilized to investigate the impact of Ag-NPs on TMV accumulation levels as well as several defense-related genes. This study examined a total of twelve genes, including five defense-related genes (*PR-1*, *PR-2*, *PR-12*, *POX*, and *GST-1*), two genes related to polyphenol metabolism (*PAL-1* and *HQT-1*), two *SbWRKY* transcription factor genes, one *JERF-3* response factor gene, and the *TMV-CP* gene (Table 1). The reference gene Elongation factor 1-α was used to normalize the expression levels. The RT-qPCR was carried out on a Rotor-Gene 6000 (QIAGEN, USA) using SYBR Green PCR master mix (Fermentas, USA). The reaction was started with initial denaturation at 95 °C for 10 min. After that, there were 40 cycles of denaturation at 95 °C for 15 s, annealing at 60 °C for 30 s, and extension at 72 °C for 30 s. Each sample was tested in triplicate. The 2^−∆∆CT^ approach was used to accurately quantify and calculate the relative expression level of the target gene [42] as follows:

ΔC_T (target)_ = (C_T (target)_ - C_T (reference)_); ΔC_T (control)_ = (C _T (control)_ - C _T (reference)_). The quantity of ΔΔC_T_ to the target gene was determined with equation: ΔΔC_T_ = (C_T (target)_) - C _T (control)_) confirmed to 2^− ΔΔCT^ algorithm.

**Table 1:** Primer sequences of RT-qPCR primers used in this study

Gene name	Abbreviation	Primer Sequence (5′–3′)	Pathway
WRKY transcription factor 1	*SbWRKY-1 F*	CGTGCAGCAGCAAAGCAA	SbWRKY transcription factors
	*SbWRKY-1R*	GTCGCAGGTATGCTCGTTGA	
WRKY transcription factor 19	*SbWRKY-19 F*	AATGTCCCTCTGGCGAACTC	
	*SbWRKY-19R*	CAGTACACCCAAGGCTCCAT	
Jasmonate and ethylene-response factor 3	*JERF-3 F*	GCCATTTGCCTTCTCTGCTTC	ET/JA-signaling pathways
	*JERF-3R*	GCAGCAGCATCCTTGTCTGA	
Glutathione S-transferase 1	*GST-1 F*	CGGTGACTTGTACCTCTTCGAATC	SA-binding receptor proteins
	*GST-1R*	ATCCACCATTGCTGCCTCC	
Peroxidase	*POD-F*	CCTTGTTGGTGGGCACACAA	SA-signaling pathway
	*POD-R*	GGCCACCAGTGGAGTTGAAA	
Pathogenesis related protein-1	*PR-1 F*	CCAAGACTATCTTGCGGTTC	
	*PR-1R*	GAACCTAAGCCACGATACCA	
β-1,3-glucanases	*PR-2 F*	TATAGCCGTTGGAAACGAAG	
	*PR-2R*	CAACTTGCCATCACATTCTG	
Plant defensin 12	*PR-12 F*	CACAGAAGTTGTGCGAGAGG	
	*PR-12R*	GCAAGATCCATGTCGTGCTT	
Phenylalanine ammonia-lyase	*PAL-1 F*	ACGGGTTGCCATCTAATCTGACA	Phenylpropanoid biosynthetic
	*PAL-1R*	CGAGCAATAAGAAGCCATCGCAAT	
Hydroxycinnamoyl-CoA quinate transferase	*HQT-1 F*	CCCAATGGCTGGAAGATTAGCTA	Chlorogenic biosynthesis
	*HQT-1R*	CATGAATCACTTTCAGCCTCAACAA	
Elongation factor 1-α	*EF1-a-F*	GAACTGGGTGCTTGATAGGC	Housekeeping
	*EF1-a-R*	AACCAAAATATCCGGAGTAAAAGA	
Tobacco mosaic virus-coat protein	*TMV-CP-F*	ATGCCATTCTCCGTCTTGACTTG	Virus replication
	*TMV-CP-R*	GAGTTGTATGTAGTCTCGTGGATT	

### Statistical analyses

All the statistical analysis was conducted on R software 4.3.2, (2023). Volcano plots were created using *ggplot2* package based on t-test to display the statistical significance (expressed as -log_10_) and gene expression (expressed as log_2_ fold-change). Heatmap and cluster analysis were performed using *pheatmap* package used log_2_ fold-change of gene expression. Gene co-expression networks (GCN) was created using *qgraph* package based on Spearman rank correlation coefficient.

## Results

### Silver nanoparticles characterization

**Fig. 1:**
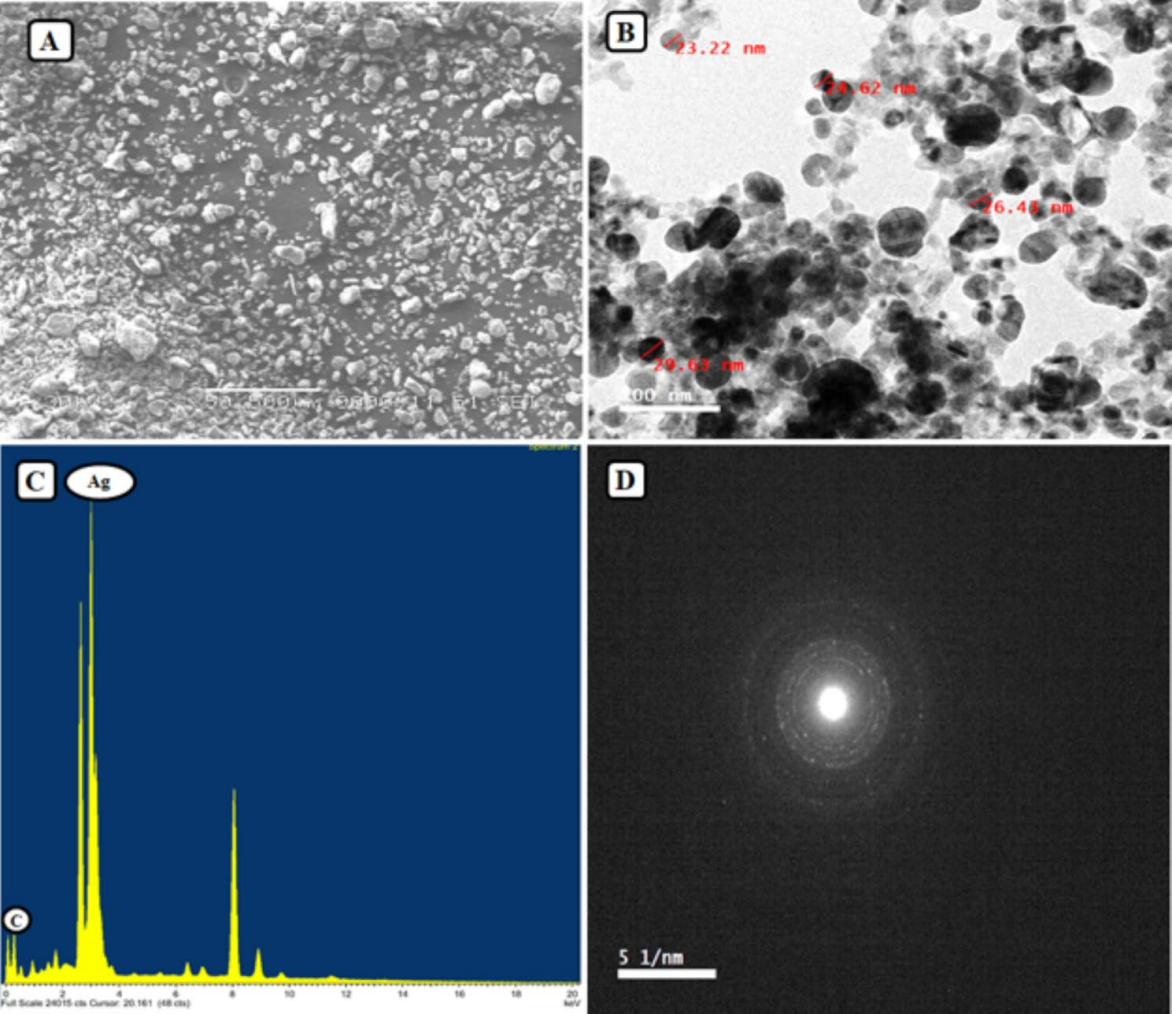
Illustration of SEM (**A**); TEM (**B**); EDS (**C**); Diffraction (**D**) analysis of silver nanoparticles biosynthesized by *Ammi visnaga* seeds extract

The SEM image of Ag-NPs showed irregular and spherical particles with an average diameter of around 50 nm (Fig. 1A). The TEM image result demonstrated that the Ag-NPs are spherical particles in shape with an average size of 23–30 nm (Fig. 1B). The EDS examination of the sample verified the elemental silver signal as the predominant element. The study of the spectrum reveals a peak in the silver area, which formally established the creation of Ag-NPs (Fig. 1C). There was another signal in the micrograph that looked a lot like carbon. It suggests that there is an organic plant seed extract there, which is the same as the biomolecules that were covering the synthesized Ag-NPs. According to Fig. 1C, an elemental analysis of the Ag-NPs shows that silver (73.91%) makes up the largest percentage, followed by the other element. Furthermore, under accurate examination, it is evident that the Ag-NPs are directly linked within the aggregates, implying the presence of a capping agent for the Ag-NPs. It can be seen that the particles are crystalline because of the ring-like diffraction pattern and the roughly circular shape of the SAED spots in Fig. 1D.

**Fig. 2:**
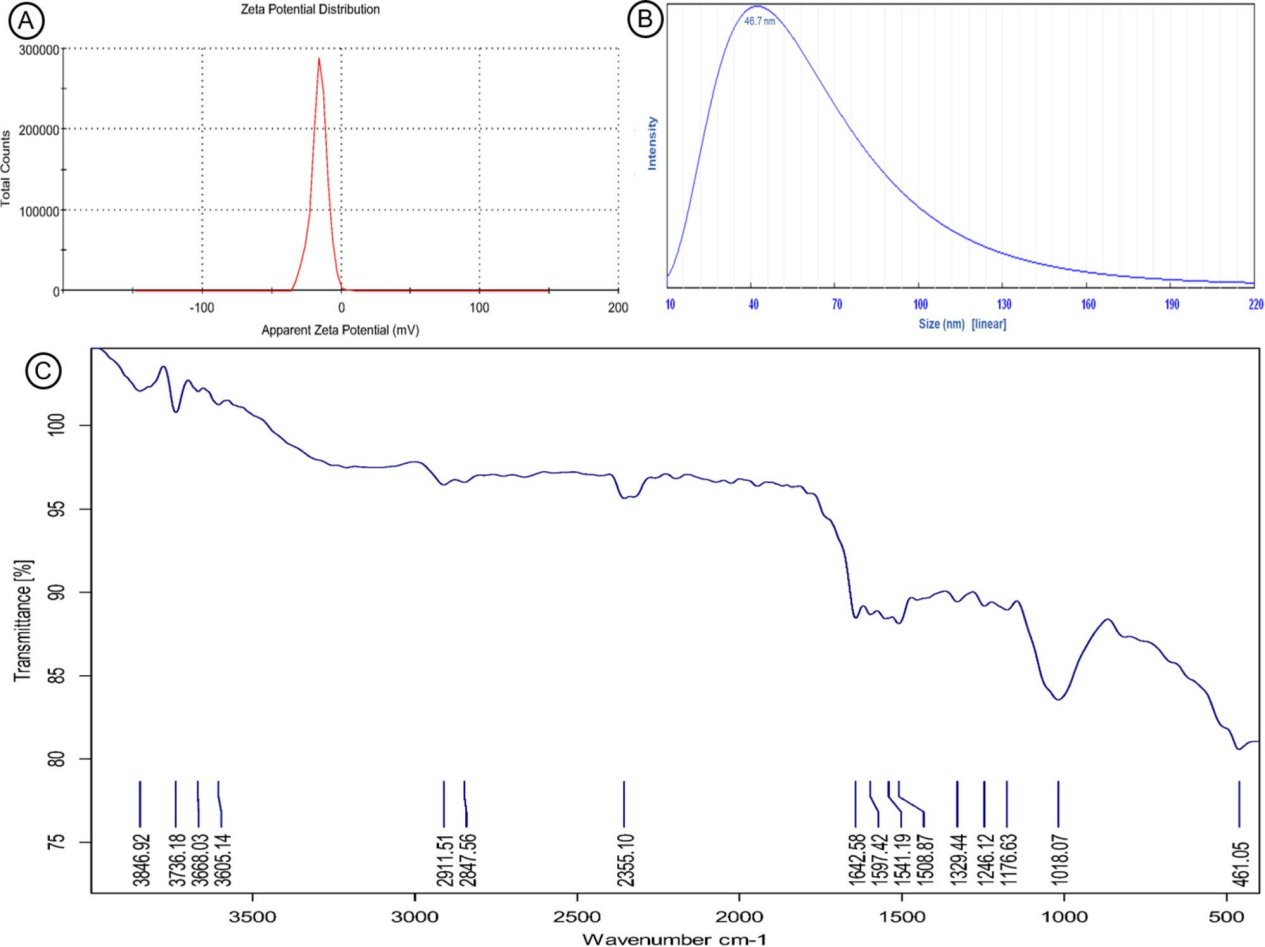
Illustration of zeta potential (**A**) DLS (**B**) and FT-IR (**C**) analysis of silver nanoparticles biosynthesized by *Ammi visnaga* seeds extract

The charge and size of the Ag-NPs were measured using a zeta potential instrument. Figure 2A depicts the size distribution profile of the environmentally friendly produced Ag-NPs. The results show that the generated Ag-NPs have a low zeta potential of -15.9 mV, indicating moderate stability. According to DLS analysis, the particle size within nanoscale, with an average of 46.7 nm (Fig. 2B). To identify the functional group in Ag-NPs, FTIR spectroscopy was used. The FTIR spectra of Ag-NPs revealed 16 distinct peaks ranging from 3846.92 cm^− 1^ to 461.05 cm^− 1^ (Fig. 2C) and Table 2. The large peak at 3846.92 cm^− 1^, indicating the existence of an inner-surface hydroxyl group. Another peak at 3605.14 cm^− 1^ indicates alcohol and phenols. The peak at 2847.56 cm^− 1^ is attributable to aliphatic compounds, while the peak at 2355.10 cm^− 1^ is due to nitriles. The peaks at 1642.58 cm^− 1^ correspond to alkenes, 1541.19 cm^− 1^, 1508.87 cm^− 1^, and 1329.44 cm^− 1^ to proteins, and 1246.44 cm^− 1^ to phosphodiesters in phospholipids. The peaks at 1176.63 cm^− 1^ and 1018.07 cm^− 1^ correspond to polysaccharides and aliphatic amines, respectively. The band at 461.05 cm^− 1^ is exclusive to alkyl halides.

**Table 2:** FTIR spectra list of band positions for Ag-NPs in this study

Wave number cm^− 1^	Band	Functional group
3846.92	O–H stretch	inner-surface hydroxyl groups
3736.18	O-H stretching	water
3668.03	OH stretch	alcohol
3605.14	O–H stretch, free hydroxyl	alcohols, phenols
2911.51	C–H stretch	alkanes
2847.56	CH and CH2 stretching	aliphatic
2355.10	C ≡ N	nitriles
1642.58	–C = C– stretch	alkenes
1597.42	N–H bend	1˚ amines
1541.19	C-N stretch	amide II band of proteins
1508.87	C-N stretch	amide II band of proteins
1329.44	C-N stretch	amide III band components of proteins
1246.44	PO-^2^stretch asymmetric	phosphodiesters groups from phospholipids
1176.63	C-O-C	polysaccharide
1018.07	C–N stretch	aliphatic amines
461.05	C–Br stretch	Alkyl halides

### Impact of Ag-NPs on symptom development and TMV accumulation level

At 18 days post-infection, the tobacco plant infected with TMV showed a higher number of local lesions and necrotic signs than the Mock plant. While in Post-1 and Post-2, a low number of local lesion symptoms were seen with an inhibition percentage of 85% when compared to TMV-infected plants alone. In addition, modest symptoms were noticed in the Pre-1 and Pre-2 treatments, with an inhibition percentage of 96% as compared to the TMV treatment. RT-qPCR assays used to measure *TMV-CP* gene accumulation showed that Pre-1 and Pre-2-treated plants had significantly lower TMV accumulation levels (5.19-fold and 3.65-fold, respectively) than TMV treatment (267.42-fold). We found that the Post-1 and Post-2 treatments reduced TMV accumulation expression levels by 31.25-fold and 29.63-fold, respectively.

### Impact of Ag-NPs on chlorophyll, flavonoid and total soluble carbohydrate content

**Fig. 3:**
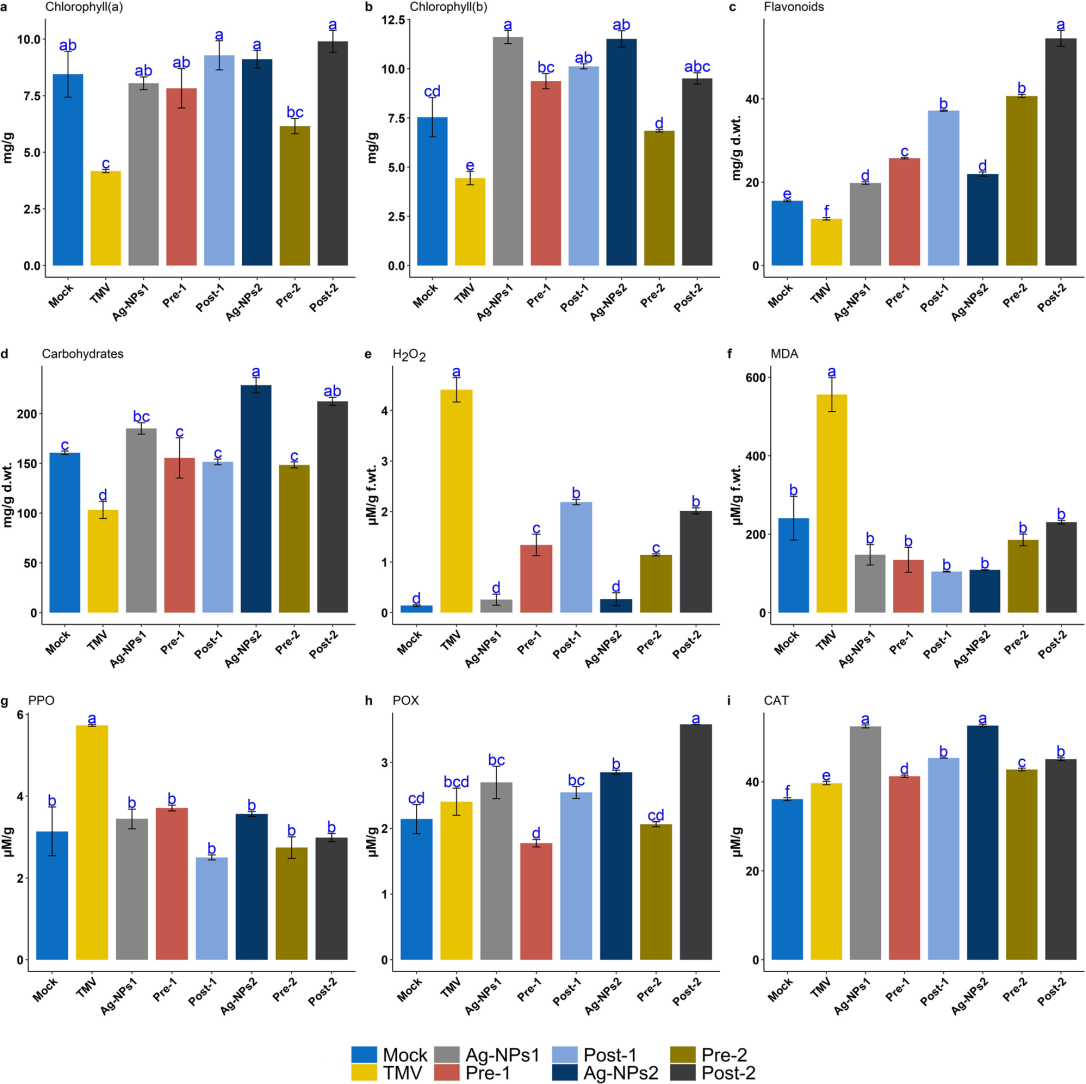
Effect of treatments on physiological traits and enzymes. Tukey’s HSD test at *p* ≤ 0.05 was used to control type I error in multiple comparisons of treatments

There was a notable increase in the levels of chlorophyll a and b in Ag-NPs-treated tobacco plants (Fig. 3a and b). The Ag-NPs1 treatment had greater values of 8.05 and 11.62 for chlorophyll a and b than the Post-1 (7.83 ± 1.51 mg/g and 9.37 ± 0.67 mg/g) and Pre-1 (9.28 ± 1.12 mg/g and 10.12 ± 0.22 mg/g), treatments, respectively. Tobacco plants treated with Ag-NPs2 solution had chlorophyll a and b contents of 9.11 ± 0.68 mg/g and 11.52 ± 0.72 mg/g, respectively. Following this came the Post-2 treatment, which had 6.16 ± 0.59 mg/g for chlorophyll a and 6.85 ± 0.11 mg/g for chlorophyll b. On the other hand, the Pre-2 treatment reported chlorophyll a and b contents of 9.9 ± 0.84 mg/g and 9.51 ± 0.49 mg/g, respectively. Moreover, the TMV treatment showed a significant decrease in chlorophyll a and b (4.17 ± 0.12 mg/g and 4.44 ± 0.59 mg/g). The TMV treatment had a lower flavonoid content (11.20 ± 0.29 mg/g d.wt.) compared to the mock plants (15.52 ± 0.21 mg/g d.wt.), which is consistent with the fact that the mock plants were better at removing free radicals (Fig. 3c). The Ag-NPs1 treatment had the same effect on flavonoid content. The Post-1 treatment significantly increased flavonoid content to 19.79 ± 0.3 mg/g d.wt., and the Pre-1 treatment significantly increased it to 37.18 ± 0.14 mg/g d.wt. The Post-2 treatment increased flavonoid levels to 40.68 ± 0.38 compared to Ag-NPs2, while the Pre-2 treatments had the highest flavonoid content at 54.53 ± 1.91 mg/g d.wt. The results (Fig. 3d) demonstrated a decrease in the total soluble carbohydrate levels in the TMV treatment (103.24 ± 8.58 mg/g d.wt.) compared to Mock plants (160.70 ± 1.78 mg/g d.wt.). The foliar application of silver nanoparticles in Ag-NPs1 and Ag-NPs2 treatments increased total carbohydrate contents by 185.16 ± 5.75 and 228.62 ± 7.76 mg/g d.wt., respectively. However, Post-1 and Post-2 treatments resulted in a slight reduction in carbohydrate contents to 151.52 ± 2.86 and 148.49 ± 3.04 mg/g d.wt., respectively.

### Impact of Ag-NPs on oxidative stress markers and antioxidant enzymes activity

The data analysis revealed that TMV infection resulted in elevated levels of H_2_O_2_ (4.41 ± 0.24 µM/g f.wt.) and MDA (556.1 ± 43.44 µM/g f.wt.) in infected leaves when compared to Mock, Ag-NPs1, and Ag-NPs2 treatments (Fig. 3e and f). The H_2_O_2_ levels were 0.26 ± 0.11 and 0.27 ± 0.13 µM/g f.wt. for Ag-NPs1 and Ag-NPs2, respectively. The MDA levels were 147.5 ± 26.14 and 108.8 ± 0.82 µM/g f.wt. for Ag-NPs1 and Ag-NPs2, respectively. The PPO analysis indicated that the treatment with TMV had the highest value at 5.73 ± 0.02 µM/g, whereas the other treatments did not exhibit any significant changes (Fig. 3g). In POX analysis (Fig. 3h), Pre-2 had a greater value (3.58 ± 0.00 µM/g) than Ag-NPs2 (2.85 ± 0.03 µM/g), whereas Pre-1 had a lower value (2.54 ± 0.09 µM/g) than Ag-NPs1 (2.70 ± 0.25 µM/g). There was no significant difference between those infected with TMV (2.40 ± 0.21 µM/g) and those treated with Post-2 (2.06 ± 0.04 µM/g). The Mock and Post-1 treatments measured 2.14 ± 0.22 µM/g and 1.77 ± 0.06 µM/g, respectively. For CAT analysis (Fig. 3i), Ag-NPs1 and Ag-NPs2 treatments achieved the highest values of 52.65 ± 0.28 and 52.47 ± 0.34 µM/g, respectively. The TMV treatment measured 39.70 ± 0.38 µM/g, while the Mock treatment had the lowest value (36.10 ± 0.31 µM/g). The Pre-1 and Pre-2 showed 41.26 ± 0.25 µM/g and 42.78 ± 0.29 µM/g, respectively, while Pos1-1 and Post-2 showed 45.34 ± 0.03 µM/g and 45.10 ± 0.30 µM/g, respectively.

### Impact of Ag-NPs transcriptional levels of defenserelated genes

The qPCR results showed that the Pre-1 and Post-1 treatments significantly boosted *PR-1* transcription levels with a 9.58- and 5.61-fold change, respectively, greater than the Mock treatment. Moreover, Pre-2, TMV, and Post-2 treatments exhibited relative expression levels of 4.87-, 4.27-, and 3.87-fold, respectively, higher than the control. Similarly, Pre-2 treatment increased the expression level of *PR-2* by 18.55-fold, followed by TMV treatment (11.77-fold) and Post-2 treatment (11.20-fold greater than the control). Both Pre-1 and Post-1 treatments exhibited 7.08 and 4.92 times, respectively. Concerning *PR-12*, it showed a considerable increase in expression of the Pre-2 treatment with a fold change of 22.92, followed by Post-2 with a 9.93-fold higher than Mock treatment. The Pre-1 and Post-1 treatments showed relative expression of 7.34- and 2.91-folds, respectively, greater than the control. Regarding *POD*, the highest relative expression levels were shown in the Pre-2 and Post-2 treatments, with 57.83- and 39.07-fold changes, respectively, higher than the control. For *GST-1*, all treatments triggered the gene expression. The Pre-1 and Post-2 treatments exhibited the highest relative expression levels of 88.07- and 41.46-fold, respectively, greater than the control. In addition, Pre-2 and Post-2 reported transcriptional levels of 22.26- and 9.83-fold, respectively.

The qPCR analysis of two polyphenol biosynthetic pathway genes, *PAL-1* and *HQT-1*, revealed the induction of *PAL-1* in all study treatments. The Ag-NPs2 Pre-2 and Post-2 treatments showed higher expression levels with 21.72-, 25.12-, and 17.40-fold, respectively. On the other hand, Ag-NPs1, Pre-1, and Post-1 exhibited transcriptional levels of 3.20-, 13.69-, and 9-fold, respectively. The TMV treatment recorded a 2.46-fold change higher than the control. The *HQT-1* gene was estimated from the chlorogenic acid pathway. The application of Pre-1 and Post-1 treatments induced the relative expression by 5.24- and 3.57-fold, respectively. Additionally, gene up-regulation was also reported in Pre-2 and Post-2 treatments with 4.40- and 2.23-fold, respectively. The TMV treatment exhibited a slight increase, with an expression level that was 1.77-fold greater than the control.

Gene expression of *SbWRKY* transcription factors (*SbWRKY-1* and *SbWRKY-19*), as well as response factor *JERF-3*. *SbWRKY-1* was up-regulated in all treatments at Ag-NPs1 and Ag-NPs2 (2.32-fold and 21.29-fold), Pre-1 and Pre-2 (26.87-fold and 23.57-fold), as well as Post-1 and Post-2 (11.60-fold and 10.98-fold). In the case of *SbWRKY-19*, Ag-NP treatments increased gene expression at pre-1 and Post-1 (8.87-fold and 2.98-fold), as well as Pre-2 and Post-2 (21.02-fold and 8.52-fold). Otherwise, the Ag-NPs1 and Ag-NPs2 treatments had no influence on *SbWRKY-19* expression when compared to TMV infection alone (9.93-fold). In tobacco plants, infection with TMV alone or treatment with Ag-NPs2 alone increased gene expression (3.63-fold and 4.72-fold) compared to untreated plants, but no effect was detected with Ag-NPs1 alone on tobacco plants where *JERF-3* expression was suppressed. Furthermore, the increase of expression in the Pre-2 and Post-2 was much greater (28.95-fold and 16.43-fold, respectively) than in the Pre-1 and Post-1 (8.30-fold and 3.96-fold, respectively).

### Statistical significance of the differential gene expression

**Fig. 4:**
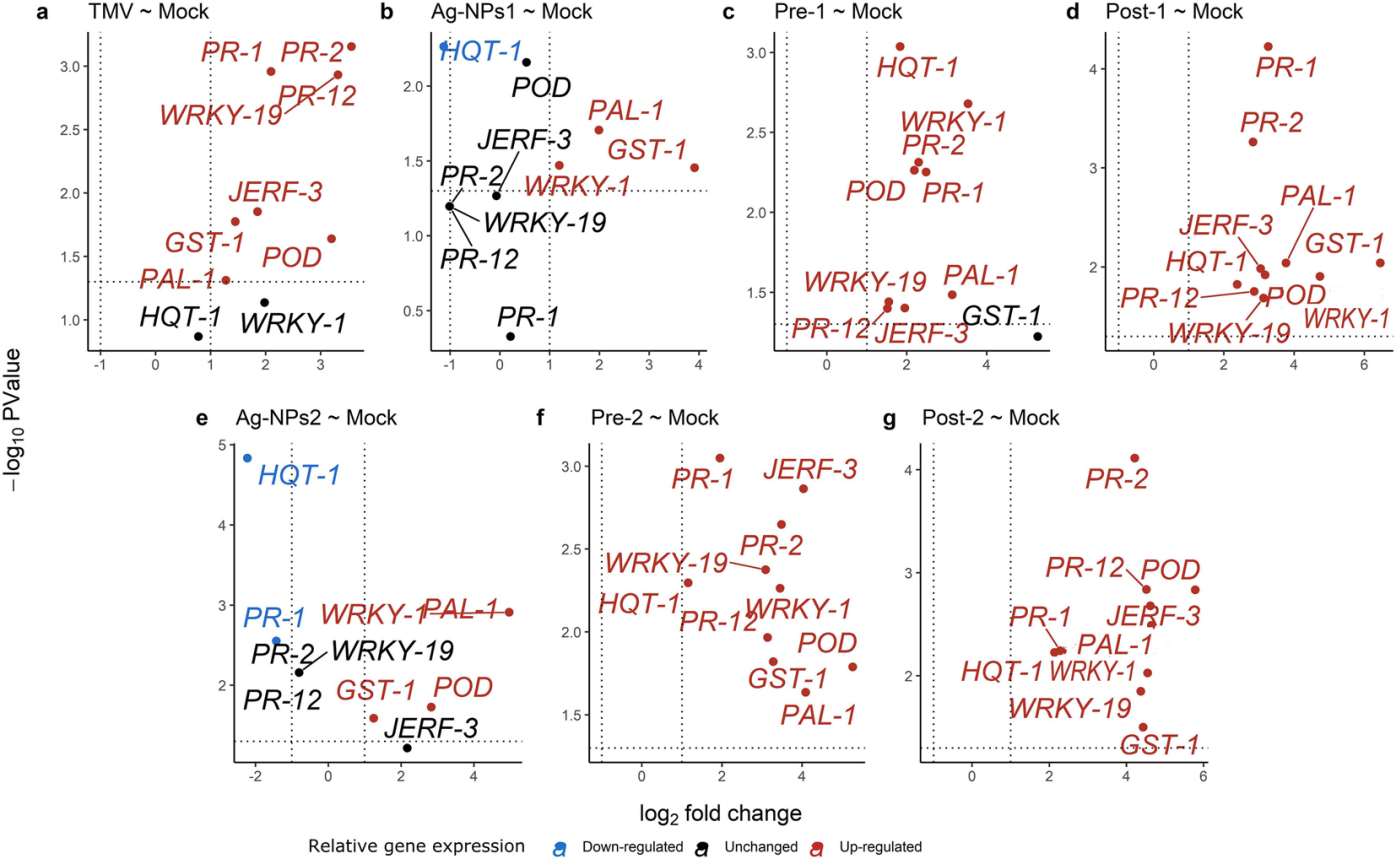
Volcano plots of gene expression, y-axis represents P-values (-log10), x-axis represents fold change (log2), genes in red color are significantly upregulated, genes in blue color are significantly downregulated, genes in black color are unchanged

Results from the volcano plots in Fig. 4a showed that the TMV treatment led to significant up-regulation for genes (*JERF-3*, *POD*, *PR-1*, *PR-12*, *PR-2*, *PAL-1*, *GST-1*, and *SbWRKY-1*9), while *HQT-1* and *WRKY-1* genes were unchanged. Treatment Ag-NPs1 (Fig. 4b) led to significant up-regulation of genes *GST-1*, *PAL-1*, and *SbWRKY-1*, while *HQT-1* was significantly down-regulated, and genes *PR-1*, *POD*, *PR-2*, *PR-12*, *SbWRKY-19*, and *JERF-3* were unchanged. For Pre-1 treatment (Fig. 4c), a total of 9 genes (*PR-1*, *HQT-1*, *PAL-1*, *PR-2*, *PR-12*, *POD*, *SbWRKY-1*9, *JERF-3*, and *SbWRKY-1*) were significantly up-regulated, while the *GST-1* gene was un-changed and no gene was down-regulated. The Post-1 treatment was significantly up-regulated for all genes (Fig. 4d). Raising the treatment level to Ag-NPs 2 (Fig. 4e) led to significant up-regulation in *SbWRKY-1*, *GST-1*, *POD*, and *PAL-1*, while 4 genes (*PR-2*, *PR-12*, *SbWRKY-1*9, and *JERF-3*) remained unchanged and 2 genes (*PR-1* and *HQT-1*) were significantly down regulated. Plants for both Pre-2 and Post-2 treatments (Fig. 4f and g) led to significant up-regulation of the 10 genes (*JERF-3*, *POD*, *PR-12*, *PAL-1*, *HQT-1*, *SbWRKY-1*, *SbWRKY-1*9, *GST-1*, *PR-1*, and *PR-2*) used in this study.

### Hierarchical clustering of the differentially expressed genes

**Fig. 5:**
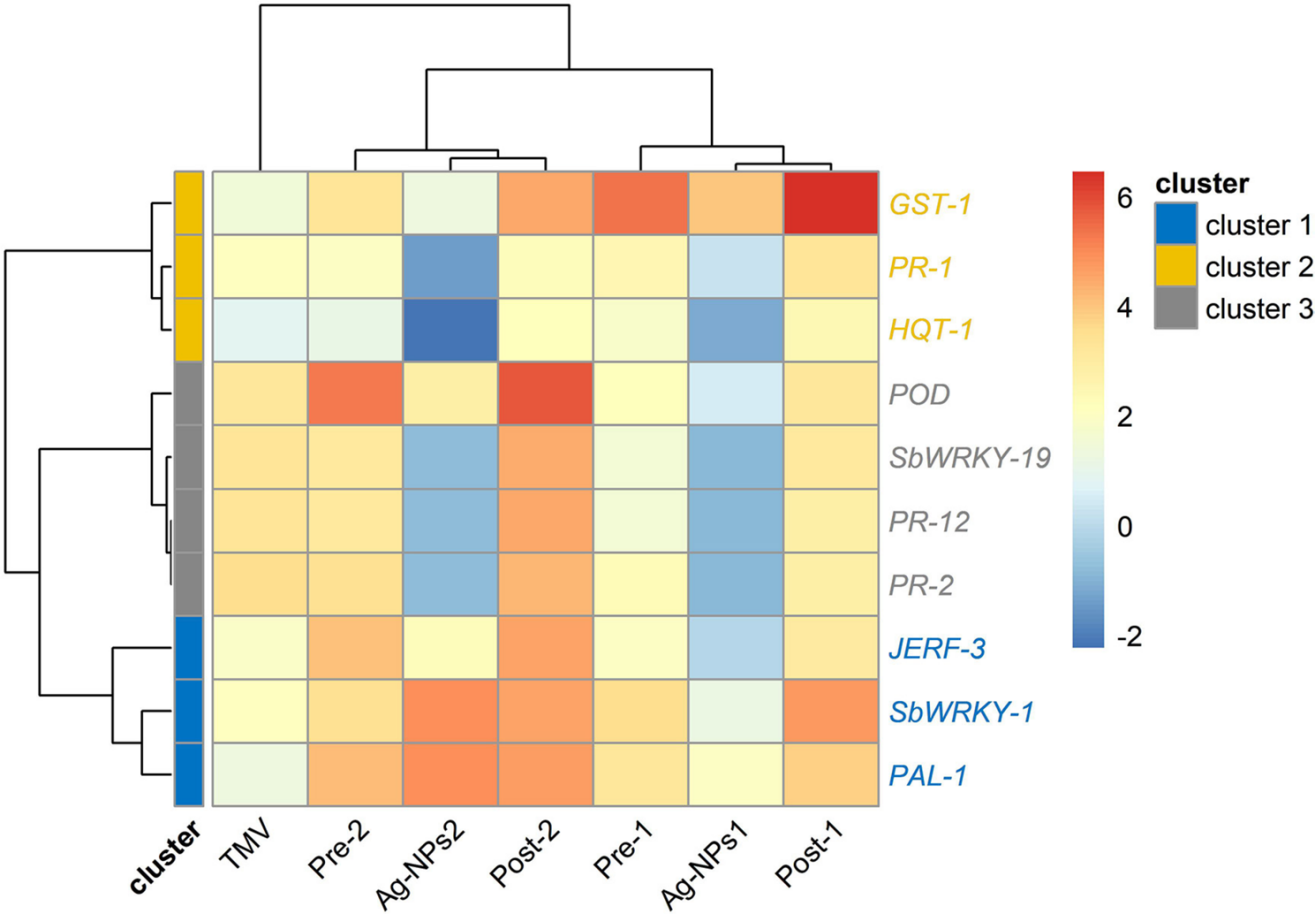
Heatmap and hierarchical clustering based on Spearman correlation-based distance and Complete method for 10 genes and 7 treatments. Cell color intensities were based on gene expression as log2 where blue color indicate downregulated genes whereas red color indicate upregulated genes

The heatmap and cluster analysis of the differential expression of the studied 10 genes are illustrated in Fig. 5. Results from the figure revealed that all studied genes were grouped into three clusters. The first cluster contained the genes *JERF-3*, *SbWRKY-1*, and *PAL-1*, while the second contained the genes *GST-1*, *PR-1*, and *HQT-1*, and the third contained the genes *POD*, *SbWRKY-1*9, *PR-12*, and *PR-2*. Two main regions (blue color) of down-regulation and unchanged genes were observed with respect to Ag-NPs1 and Ag-NPs2 treatments. In the same context, a region of up-regulation (red color) was noticed with respect to Pre-1 and Pre-2 treatments.

### Correlations between the studied genes

**Fig. 6:**
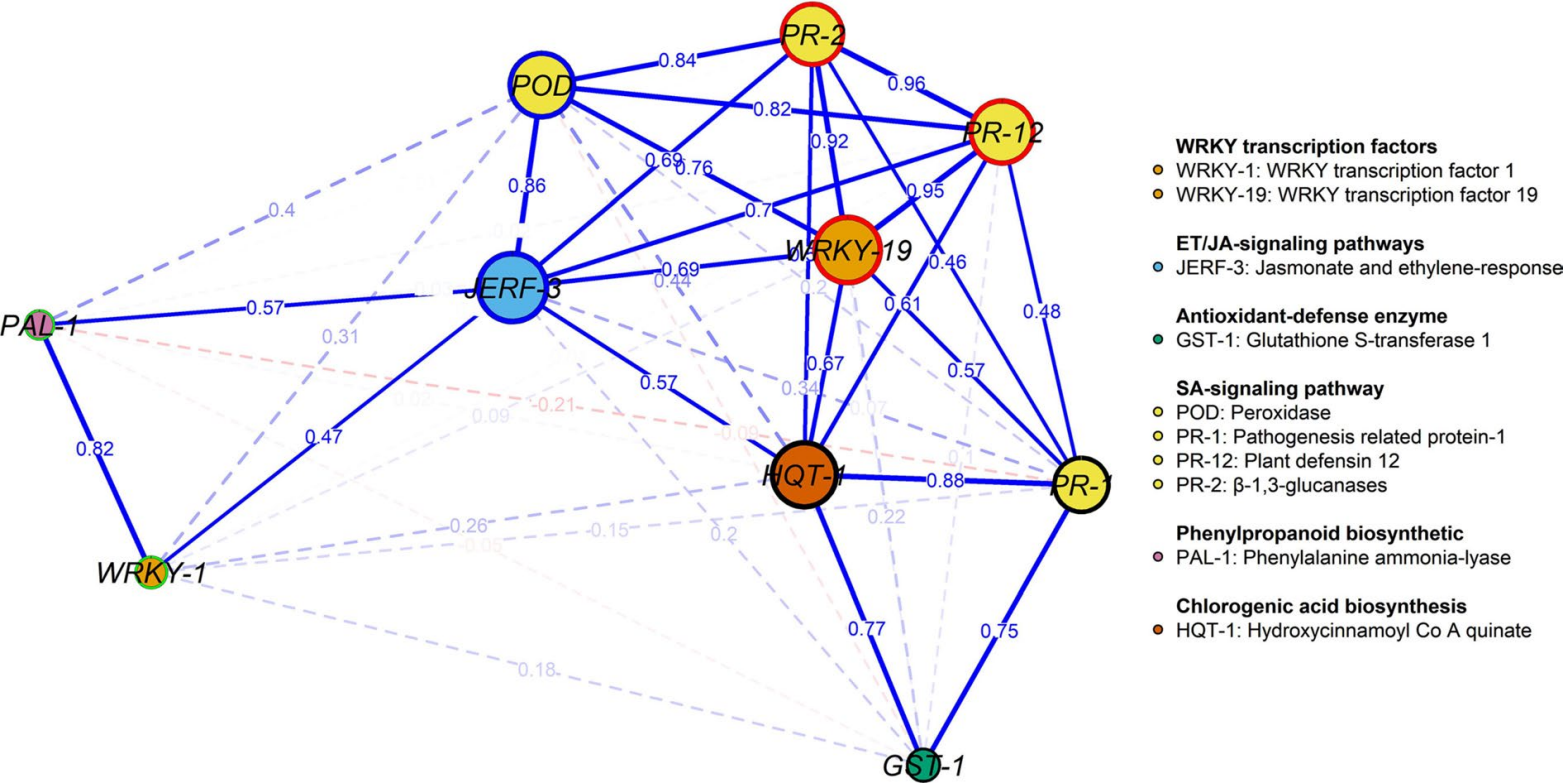
Co-expression network of the studied 10 genes, nodes (circles) represent genes and edges (lines) represent association among genes based on Spearman rank correlation

The gene co-expression network of 10 genes was made to show how the co-expression pattern changes when the TMV and Ag-NPs are used together (Fig. 6). The network revealed that genes *PAL-1*, *GST-1*, and *SbWRKY-1* were the least central genes compared to the other 7 genes. This means that they had the least number of correlations with the other 7 genes, which indicates their relative independence. An interesting result in the gene co-expression network is the appearance of local communities, where genes *PR-2*, *PR-12*, and *SbWRKY-1*9 were grouped in one community (nodes with a red border). This means that these 3 genes had a significant interrelationship with each other more than with their relationship with the other genes. Consequently, they had similar expression patterns and a high probability of sharing the regulation mechanisms for their expression. The same situation was found for genes *POD* and *JERF-3* as well as for genes *HQT-1*, *PR-1*, and *GST-1* and genes *PAL-1* and *SbWRKY-1*.

### Regression analysis

**Fig. 7:**
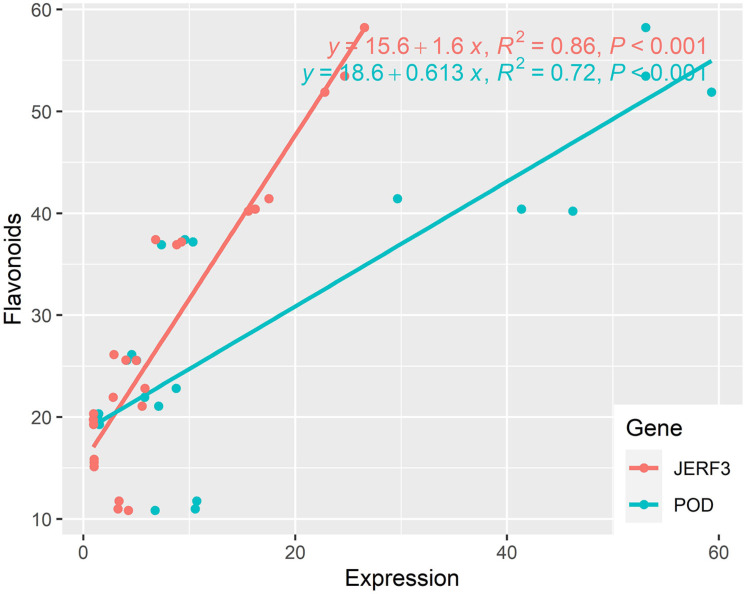
Regression analysis between JERF-3 and POD genes and Flavonoids

Figure 7 revealed that both *JERF-3* and *POD* had significant effects on flavonoids, where R^2^ was 0.86 and 0.72, respectively. It is apparent that the slopes of the regression lines of the two genes were significant; however, the effect of *JERF-3* was more robust as the value of slope and R^2^ were higher than that of *POD*. In addition, to reach the maximum level of flavonoids, it required an expression of less than 25 (x-axis) of *JERF-3*, while to reach the same level of flavonoids, it required an expression of more than 65 (x-axis) of *POD*.

## Discussion

Plant pathogens, particularly viruses, cause significant agricultural losses and pose a threat to global food security. It is critical to find and develop new biocontrol agents that can manage plant viral infections as soon as possible because of the complexity of the problem and the fact that their environment is always changing [43]. Nanoparticle treatments are critical in agriculture for lowering biotic and abiotic stresses because they either increase antioxidant production or inhibit the formation of ROS. Nanoparticles are widely used because of their small size, which allows them to flow easily via narrow membrane channels. Nanoparticles have shown promise as an antiviral agent, but much remains unknown about their mode of action and the state of their research and development. The mechanisms may involve entire or partial structural changes to the virus particle, as well as suppression of virus reproduction. These approaches differ depending on the size and qualities of the nanoparticles used. Nanoparticles commonly alter the structure of the viral nucleocapsid, reducing infectivity. Furthermore, antiviral activity is achieved indirectly by changing the permeability of the cell membrane, which prevents virus penetration [44].

In the current study, the efficacy of using *Ammi visnaga* aqueous seed extract for Ag-NPs synthesis and their potential to combat TMV were evaluated. Surface charge, typically indicated by zeta potential, is a crucial factor in determining the physical stability of nanoparticles [45]. It was reported that the highly stable nanoparticle had zeta potentials greater than 30 mV or less than − 30 mV [46]. Thus, in our study, the zeta potential value of the prepared silver nanoparticles (-15.9 mV), suggested that it had moderate stability [47]. Ag-NPs with a net negative charge were prepared using *A. visnaga* seed extract. This could be because negative functional groups (COO-, CO-, and OH-) were introduced to their surface. Nanoparticles with a negative zeta potential exhibit greater cellular absorption than those with a positive zeta potential. Nanoparticles with decreased negative charge exhibit enhanced cellular entry and interaction with cellular membranes [48]. Therefore, increasing the strength and characteristics of the electric charge surrounding the particle’s double layer can prevent the nanoparticles from clumping together. This, along with the stabilizing effect of the coating agents that have the same charge, contributes to the improved stability of the nanoparticles [49]. FTIR spectra of Ag-NPs revealed 16 distinct peaks ranging from 3846.92 cm^− 1^ to 461.05 cm^− 1^. FTIR plays a crucial role in determining chemical components such phenolic, phenolic acids, and alkaloidal functional groups that are responsible for stabilizing and synthesizing nanoparticles [10, 50, 51].

Under greenhouse conditions, the foliar applications of Ag-NPs increased chlorophyll content. Several studies found that using Ag-NPs improved the shoot and root systems, the length and width of leaves, the amount of chlorophyll, carbohydrates, proteins, and antioxidant enzymes in bean, wheat, *Brassica juncea* and lemon [52, 53]. Moreover, the accumulation of *TMV-CP* gene reduced in all Ag-NPs treatments and different concentrations compared with TMV infected plants. Similarly results with Zhang et al. [54] who reported that the accumulation is consistent with the TMV group’s growth data, symptoms, and infection. Moreover, foliar application of Ag-NPs at 50 ppm effectively suppressed of sunhemp rosette virus infection on cluster bean plants [55]. Viruses accumulating inside host cells can be used to assess the severity of an infection [56]. In addition, a prior study found that the administration of Ag-NPs 24 h after viral infection decreased its accumulation level and disease incidence [57]. Ag-NPs enter plant cells and begin their antiviral activity by disrupting viral vectors or cellular components, which stops the virus from replicating. The activation of RNA or DNA processes is how this is accomplished [58]. Ag-NPs have been demonstrated to attach to the viral genome, thereby suppressing polymerase activity and halting viral replication [59].

Plant polyphenolic compounds, including phenolic and flavonoid molecules, are recognized for their significant role in helping plants protect themselves from both biotic and abiotic stressors [60, 61]. The current investigation found that the foliar application of Ag-NPs as a protective or curative treatment enhanced flavonoid and total soluble carbohydrates, consistent with earlier studies [62, 63]. Higher levels of ROS are a common feature of a viral plant infection [64]; hence, assessing ROS levels is closely linked to the severity of the disease. The research findings indicated elevated levels of oxidative stress indicators (H_2_O_2_ and MDA) in the TMV-infected plants. These results align with findings from numerous other studies on viral infections in plants [50, 65, 66]. Elevated levels of oxidative stress indicators may be a defensive mechanism against the infection. Imbalanced ROS levels result in the oxidation of essential cell components such as DNA, proteins, and unsaturated fatty acids, leading to the deterioration of plant cells and eventual infection [67, 68]. Antioxidant enzymes are known for their capacity to protect plants from oxidative damage [69]. The key components involved in protecting against oxidative damage, CAT and POX, were activated in plants exposed to Ag-NPs. Activating superoxide dismutase, ascorbate peroxidase, and catalase enzymes in FeS-NP- and MnS-NP-nano-primed seeds made the rice seedlings healthier than those in the control group [70]. POX is essential for fighting lipid peroxidation, preserving cell membrane integrity, and maintaining cellular redox balance [71]. Antioxidant enzyme levels were found to be enhanced in plants treated with TMV and Ag-NPs in the study. The application of Ag-NPs to plant leaves stimulates the release of ROS in plant tissues, resulting in increased antioxidant enzyme activity and total soluble protein levels in response to viral infections [72–74].

Plant-induced resistance can be divided into two main routes. SAR, triggered by plant pathogens or chemical agents and controlled by salicylic acid, is the first route. This pathway in the resistance mechanism primarily involves PRs and other disease resistance proteins [8]. Induced systemic resistance (ISR) is another important pathway. It is boosted by plant microorganisms that help plants grow and is controlled by jasmonic acid and ethylene [75]. This makes plants more resistant to infection and better able to defend themselves. Moreover, plant resistance to viral infection may be associated with both processes [76]. Regarding our results for *PR-1*, *PR-2*, and *PR-12* genes, the TMV, Pre- and Post-treatments recorded the highest value. The results align with previous research indicating that Ag-NPs increase the expression of *PR-1* even during viral infection. Ag-NPs can trigger SAR pathways, the main defense mechanism in tomato plants, when exposed to TMV [10, 63]. Aseel et al. [50] found that PR-2 was up-regulated in PVY-infected plants compared with the control. Also, similar findings demonstrated that viral infections in Arabidopsis clearly induced *PR-2* [77]. Like other defense-related genes, *POD* and *GST-1* showed an increase in the Ag-NPs treatments either before or after the infection. The increased activity of *POD*, an alternative ROS producer, was linked to improved plant resistance to infection [78, 79]. The *GST-1* gene encodes an antioxidant-defense enzyme that is involved in the detoxification process of foreign substances by interacting with glutathione [80]. Appearance of slightly viral symptoms was linked with a significant 20-fold rise in defense-related genes such as *GST*, chitinase, and *PR-1* [81]. Additionally, in *A. thaliana* plants that are susceptible to beet severe curly top virus, the up-regulation of GSTs during infections could be attributed to the silencing suppressor activity of the invading virus [82]. Decreasing two particular *GSTs* in the latter phases of maize virus infection indicates that *GSTs* might contribute to developing virus resistance at the beginning of the infection [83]. Furthermore, Ag-NP treatments were found to induce two polyphenolic biosynthesis pathway genes (*PAL-1* and *HQT-1*). *PAL-1* serves as a marker for induced resistance in many plant species. Enhancing the expression of the *PAL-1* gene can increase plant resistance to disease [84]. Plants that were treated with nanoclay before being infected with PVY had higher levels of *PAL-1*. This shows that *PAL-1* is an important part of plants’ defense systems that helps build chemical and structural barriers against pathogens [50]. An important transferase enzyme involved in the production of chlorogenic acid is encoded by this *HQT-1* gene. In addition to describing different pathogen resistance pathways, some investigations also documented the critical function of chlorogenic acid as an antioxidant molecule [85, 86]. Further, Pre and Post treatments enhanced *HQT-1* gene expression compared to control plants. The study showed that using Ag-NPs preventatively can enhance *HQT-1* gene expression, possibly because to the buildup of phenolic compounds and the initiation of SAR against viral infections [41, 87].

Plants use cellular signaling pathways to trigger transcriptional cascades that help them overcome the challenges they confront. The protective gene-regulatory networks are activated when these pathways and signaling molecules work together [88]. *WRKY* proteins play a crucial role in regulating transcription networks and setting off plant responses to both biotic and abiotic stressors [89]. *WRKY* transcription factors associated with W-boxes are present in stress-responsive promoters of various plant defense genes. Clusters of W-boxes indicate coordinated interactions among numerous *WRKY* transcription factors [90]. *WRKY-1* regulates the SA signaling pathway by interacting with the *NPR-1* gene (Natriuretic Peptide Receptor 1). The *NPR-1* gene plays a crucial role in coordinating plant defensive responses by controlling the expression of more than 2000 defense-related genes [91, 92]. Increased seed germination and disease resistance are the result of a series of synergistic events in NP-primed seeds, which include improved metabolism, stimulation of hormone secretion, modulation of biochemical signaling pathways, reduction of reactive oxygen species, reduction of reactive oxygen species, and upregulation of aquaporin gene expression [93]. *JERF-3* interacts with the GCC box in the promoters of several defense-related genes, initiating various defense mechanisms [94]. It is an important part of the ET/JA signaling pathways. Two *SbWRKY* transcription factors (*SbWRKY-1* and *SbWRKY-19*) and the response factor *JERF-3* were assessed in this work. They exhibited higher levels in plants treated with Ag-NPs prior to TMV infection, acting as a defense mechanism for the plants. Importantly, foliar treatment of Ag-NPs has been shown to induce resistance to TMV in tobacco by inhibiting viral replication and viral movement. The *WRKY* transcription factor has been implicated in plant viral defense mechanisms. *WRKY-1* and *WRKY-3* were involved in N gene-mediated resistance to TMV in *N. benthamiana* plants [95]. On the other hand, *WRKY-8* plays a role in the antiviral response against crucifer-infecting TMV by limiting the long-distance movement of TMV in Arabidopsis [96]. Although *WRKY* TFs play a crucial role in plant resistance, their involvement in plant antiviral defense is not as well understood as their role in defense against fungal and bacterial diseases.

## Conclusion

The Ag-NPs generated using an aqueous extract of *Ammi visnaga* had a spherical shape, an average size of 25 nm, and exhibited good stability with a zeta potential of -15.9 mV. FTIR analysis identified different functional groups responsible for the stability and capping of Ag-NPs. Under greenhouse conditions, the treatment of tobacco plants with Ag-NPs at 100–500 µg/mL either before or after TMV inoculation results in a significant reduction of symptoms appearance and delays. Also, treatments with Ag-NPs greatly increased chlorophyll a and b, total flavonoids, total soluble carbohydrates, and antioxidant enzymes. Oxidative stress markers also decreased. The RT-qPCR and volcano plots showed that treatments with Ag-NPs activate and regulate ten genes that are involved in defense. The heatmap showed that *GST-1* was the main gene involved in anthocyanidin synthesis, while the gene co-expression network reported that *SbWRKY-19* was the most important gene. Consequentially, we advocate employing these nanoparticles in pest management programs to fight plant virus diseases. However, further application under field conditions is required.

## Data Availability

All data generated or analyzed during this study is included in this manuscript and is available from the corresponding author upon reasonable request.
